# A case report of esophageal heterotopic pancreas presenting as recurrent mediastinal abscess, treated by minimally invasive esophagectomy

**DOI:** 10.1016/j.ijscr.2019.09.044

**Published:** 2019-10-23

**Authors:** Edno Tales Bianchi, Francisco Tustumi, André Fonseca Duarte, Evelin Sánchez Ortiz, Sérgio Szachnowicz, Francisco Carlos Bernal da Costa Seguro, Rubens Antônio Aissar Sallum, Ivan Cecconello

**Affiliations:** Department of Gastroenterology, Digestive Surgery Division, University of São Paulo Medical School, Av. Dr. Enéas de Carvalho Aguiar 255, Sao Paulo, SP, 05403-000, Brazil

**Keywords:** Esophageal neoplasms, Esophagectomy, Pancreatitis, Accessory pancreas

## Abstract

**Introduction:**

Heterotopic pancreas is a rare congenital anomaly. We report a case of esophageal heterotopic pancreas complicated by recurrent mediastinal abscess and treated by minimally invasive resection.

**Presentation of case:**

A 31-year-old woman was admitted with a history of recurrent chest pain, dysphagia, and heartburn. CT scan revealed focal confined collection in the lower mediastinum surrounding esophagus. Endoscopic ultrasound revealed a subepithelial lesion. The patient was treated by minimally invasive esophagectomy and made an uneventful postoperative recovery.

**Discussion:**

The management of subepithelial lesions would depend on their size, ability to exclude other etiologies and their associated symptoms. The patient, in this case, was obviously symptomatic and accurate differentiation from malignant etiologies could not be accurately made.

**Conclusion:**

Although pancreatic heterotopia is rare, it should be remembered in the differential diagnosis of various gastrointestinal lesions.

## Highlights

• Heterotopic pancreas is a rare congenital anomaly.
• Patients may present with complications such as inflammation and abscess.
• The management depends on size, ability to exclude other etiologies and symptoms.

## 1 Introduction

Heterotopic pancreas, also known as ectopic, or accessory pancreas, is defined as pancreatic tissue outside the normal pancreatic parenchyma with a vascular and nerve supply separate from the pancreas itself [1]. It is quite an unusual anomaly and often an incidental asymptomatic finding with no clinical significance [2,3]. It is thought that heterotopic pancreas derivatives from the primitive foregut, during the separation of the pancreatic tissue buds in the fetal development [[4], [5], [6]]. Most prevalent anatomic sites are stomach, duodenum, and jejunum [2]. Few cases of esophageal heterotopic pancreatic tissue have been reported [[7], [8], [9], [10], [11], [12]].

Complications of the heterotopic pancreas are pancreatitis of the heterotopic tissue, pseudocyst formation, abscess, endocrine dysfunction, malignant degeneration, mechanical obstruction, and bleeding [4,6,[13], [14], [15]].

We report an exceedingly rare case of symptomatic esophageal heterotopic pancreas appearing as recurrent mediastinal abscess, and treated as minimally invasive esophagectomy. The work has been reported in line with the SCARE criteria [16].

## 2 Presentation of case

A 31-year-old black woman was admitted with a history of recurrent chest pain, dysphagia, and heartburn, no complaint of fever or weight loss, and no significant past medical history. Patient denied swallowing any foreign body. She denied alcohol consumption. On admission, physical examination revealed normal sinus rhythm, normal pulse and blood pressure, normal temperature, and mild abdominal pain, without peritonitis. BMI: 22.1 kg/m^2^.

**Fig. 1:**
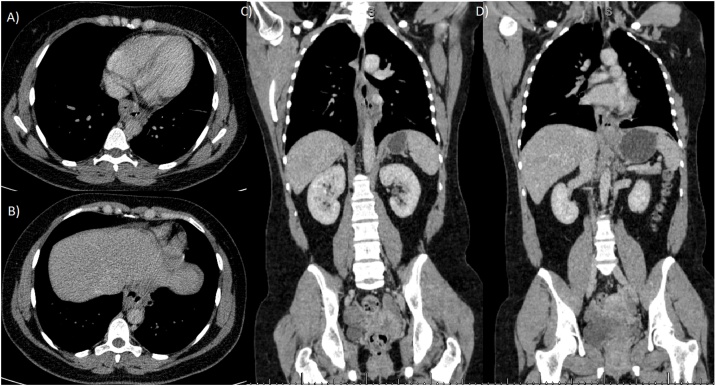
Computed tomography. A and B: axial view, showing a confined collection with air in the lower mediastinum surrounding esophagus. C and D: coronal view showing a confined periesophageal collection, and distal esophageal wall thickening.

**Fig. 2:**
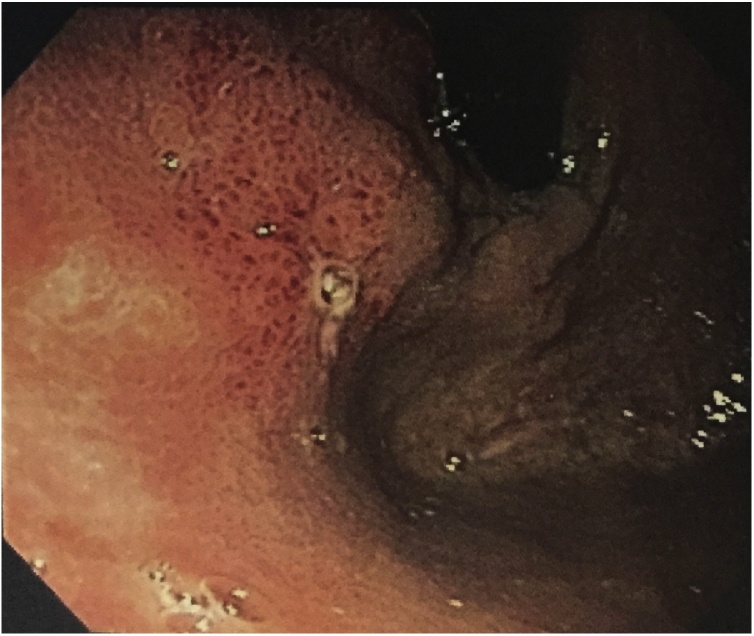
Endoscopy. Endoscopy showed reddish budging of the cardia mucosa with purulent drainage orifice.

**Fig. 3:**
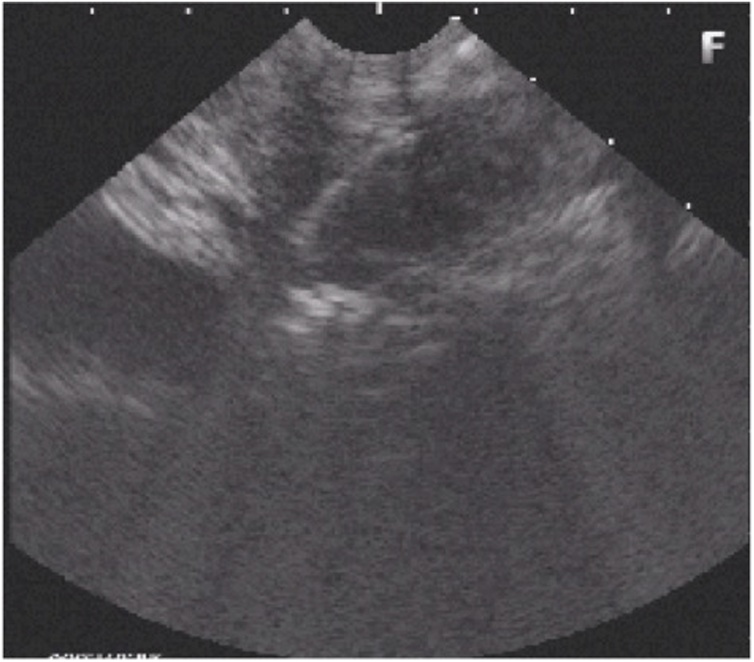
Endoscopic ultrasound. Endoscopic ultrasound showed a 31.5 × 9.1 mm subepithelial anechoic oval lesion in distal lateral esophageal wall, 37 cm from incisors.

Lab results: Hgb: 14.4 g/dL; WBC: 9.3 × 103/μL; serum amylase: 653 U/L; serum lipase: 544 U/L; liver enzymes were within normal limits. CT scan revealed distal esophageal wall thickening and focal confined collection with air in the lower mediastinum surrounding esophagus, and pancreas showed neither parenchymal enlargement nor changes in density (Fig. 1). Endoscopy was performed, showing reddish budging of the cardia mucosa with purulent drainage orifice (Fig. 2). Endoscopic ultrasound revealed a 31.5 × 9.1 mm subepithelial anechoic oval lesion in distal lateral esophageal wall, 37 cm from incisors (Fig. 3). The fine-needle aspiration punction revealed only a few epithelial cells. The patient was treated with antibiotics and fasting, with the resolution of infection.

The main differential diagnoses of subepithelial esophageal lesions are leiomyoma, lipoma, varices, neural tumors (i.e., schwannoma, neuroma, or neurofibroma), granular cell tumor, inflammatory fibroid polyp, duplication cyst, lymphangioma, Brunner’s gland hyperplasia, GIST, carcinoid, and metastatic carcinoma [17]. After comprehensive imaging and repeated biopsies, the differential diagnosis remained unclear, although age and subsequent follow-up endoscopic ultrasound imaging argue for benign disease.

**Fig. 4:**
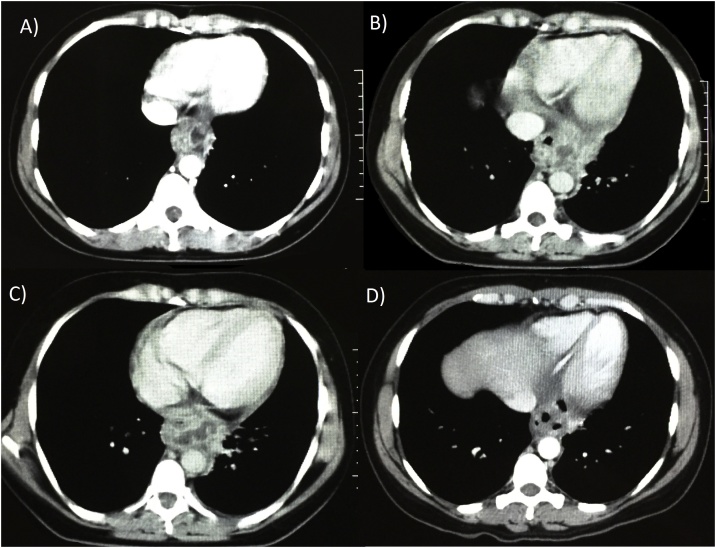
Computed tomography. Along two-year follow-up, the patient presented four (A–D) episodes of mediastinal abscesses.

Along two-year follow-up, the patient had four readmissions with a similar clinical presentation (Fig. 4), all of which were treated with antibiotics and fasting. After infection resolution of the last episode, the patient was submitted to a minimally invasive McKeown esophagectomy (video 1) to avoid recurrence of symptoms. Surgery was carried out via right thoracoscopic approach and laparoscopy by a team of specialized esophageal surgeons. The patient was positioned in the semi-prone position after induction anesthesia and selective bronchial intubation. Esophageal dissection was somewhat challenging due to the surrounded fibrotic tissue following repeated inflammation of adjacent structures. The subepithelial lesion was not noted during surgery. A gastric tube was made with a laparoscopic stapler, and a mechanical cervical anastomosis was performed.

**Fig. 5:**
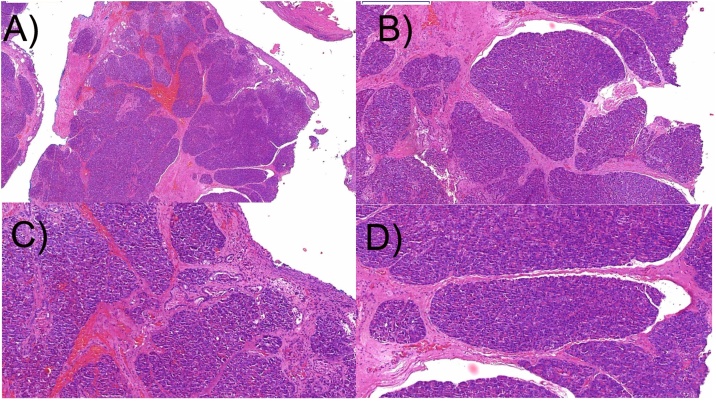
Haematoxylin & eosin (HE) stained tissue section cut. Heterotopic pancreatic tissue composed of acini and ducts, with no islet cell, within an ulcerated cyst wall. A: 1000 μm; B: 200 μm; C and D: 200 μm.

The patient made an uneventful postoperative recovery, being discharged from hospital 10 days after surgery. Pathological analysis of specimens revealed a heterotopic pancreatic tissue, containing acini, ducts, without islet cells (Fig. 5).

After a two-year follow-up, the patient had no complaints, with no recurrent symptoms.

## 3 Discussion

Esophageal heterotopic pancreas with recurrent inflammation and abscess formation treated by minimally invasive esophagectomy has not been previously reported. Lowry et al. [8] and Takemura et al. [11] reported thoracoscopic resection of the heterotopic pancreas; and Gananadha et al. [7] and Garn et al. [18] reported heterotopic pancreas laparoscopic resection in esophagogastric junction, with partial fundoplication. All of these authors performed either an extra-mucosal resection or only a short-segment esophageal/esophagogastric junction resection.

In this case report, an extra-mucosal resection would be risky due to the high level of surrounded fibrotic tissue, besides the fact that the possibility for malignant lesion was not ruled out, yet unlikely. Also, short-segment distal esophageal resection may be associated with severe gastroesophageal reflux [19]. Another issue associated with short-segment distal esophageal reconstruction is that an anastomosis would be placed in an intrathoracic position. If a mediastinal leak develops, the consequences may be more devastating than those resulting from a cervical leak [19].

The management of subepithelial lesions would depend on their size, ability to exclude other etiologies, and their associated symptoms. The patient, in this case, was obviously symptomatic and accurate differentiation from malignant etiologies could not be accurately made. Endoscopic ultrasound is the most accurate study to differentiate submucosal lesions, especially with ultrasound-guided fine-needle aspiration [17]. In this case report, multiple biopsy specimens were non-diagnostic, and imaging studies were inconclusive, hindering preoperative diagnosis. Thus, both the patient and the multidisciplinary team agreed on esophageal resection.

## 4 Conclusion

Heterotopic pancreas is an uncommon congenital anomaly. While the majority of patients are asymptomatic, patients may show clinical complications such as inflammation and abscess. We report a case of esophageal heterotopic pancreas complicated by recurrent abscess treated by minimally invasive resection. Although pancreatic heterotopia is rare, it should be remembered in the differential diagnosis of various gastrointestinal lesions.

## Funding

The authors received no specific funding for this work.

## Ethical approval

Ethical approval exemption was given for this study.

## Consent

Written informed consent was obtained from the patient for publication of this case report and accompanying images. A copy of the written consent is available for review by the Editor-in-Chief of this journal on request.

## Registration of research studies

Not applicable.

## Guarantor

Francisco Tustumi.

Edno Tales Bianchi.

## Provenance and peer review

Not commissioned, externally peer-reviewed.

## Declaration of Competing Interest

The authors declare no conflict of interest.

## CRediT authorship contribution statement

**Edno Tales Bianchi:** Conceptualization. **Francisco Tustumi:** Writing - original draft. **André Fonseca Duarte:** Writing - review & editing. **Evelin Sánchez Ortiz:** Methodology, Writing - original draft, Writing - review & editing. **Sérgio Szachnowicz:** Methodology. **Francisco Carlos Bernal da Costa Seguro:** Formal analysis, Investigation. **Rubens Antônio Aissar Sallum:** Validation, Supervision. **Ivan Cecconello:** Supervision.
